# Differential Balance Enhancements Associated with Conventional Balance Training and Portable Slackline Board Training: A Randomized Controlled Trial

**DOI:** 10.3390/sports13120457

**Published:** 2025-12-18

**Authors:** Trent Yamamoto, Joshua A. Kidwell, Vishruth Shatagopam, Kyle J. Hetherton, Eric V. Neufeld, Brett A. Dolezal

**Affiliations:** 1 Chobanian and Avedisian School of Medicine, Boston University, Boston, MA 02118, USA; 2 UCFIT Digital Health-Exercise Physiology Research Laboratory, University of California, Los Angeles, CA 90095, USA; 3 School of Medicine, Creighton University, Omaha, NE 68124, USA; 4 Northwell Orthopedics, New Hyde Park, NY 11042, USA; 5 Long Island Jewish Medical Center, North Shore University Hospital, New Hyde Park, NY 11030, USA

**Keywords:** American football, static and dynamic balance, mSEBT, sway path length

## Abstract

Compromised balance is implicated with increased injury risk in athletes. Although multiple modalities are available to improve balance, investigation into slackline balance training (SLT) has remained limited, especially in regard to elite athletes. The primary aim of this study was to determine the effects of SLT, using a novel portable slacklining board, on static and dynamic balance. Thirty male NCAA Division I American football athletes were randomized into one of three groups: a control group (CON) who received no balance training, a group undergoing conventional balance training (CBT), or SLT. Each group trained thrice weekly for eight weeks. Body mass, in addition to sway path length on three surfaces and the modified Star Excursion Balance Test (mSEBT), was assessed before and after the training period. Improvements in static and dynamic balance were observed in CBT and SLT compared to CON (*p* < 0.05). Notably, superior balance enhancements were observed in SLT relative to CBT in wobble board sway path length (*p* < 0.031), posteromedial mSEBT performance (*p* < 0.05), and composite mSEBT scores (*p* = 0.033). These results are the first to suggest that SLT may confer balance benefits in elite American football players that are comparable and, in some conditions, superior to those associated with CBT.

## 1. Introduction

Balance is a vital component of performing a wide spectrum of physical activities, ranging from routine acts of daily living to sport-specific actions. Defined as the ability to maintain one’s center of gravity within their base of support [1], balance has far-reaching implications across health and fitness contexts. Efforts to improve balance have been facilitated through a variety of training modalities, such as tilt boards [2], wobble boards [3], and Bosu balls [4]. By creating opportunities for individuals to practice responding to instability, this equipment allows individuals to improve their balance. However, the application of these modalities and their respective training programs is highly dependent on age and intended training goals.

For younger adults and athletes, balance training has a functional, rather than reactionary, emphasis. Athletes tend to incorporate balance-oriented exercises into their training in order to decrease the likelihood of injury while improving athletic performance in a variety of sport disciplines, such as gymnastics, basketball, and soccer [5,6]. In healthy, college-aged adults, balance training is often facilitated with the intention of combating chronic ankle instability (CAI) through proprioceptive training [7,8]. Stretching and strengthening muscles around the ankle, including those involved in plantarflexion, dorsiflexion, inversion, and eversion, is believed to improve balance while reducing the injury risk associated with CAI [9]. In this context, wobble board training (WBT) in particular has demonstrated promising results. Following a four-week period of WBT, significant improvements have been observed in metrics corresponding to static and dynamic balance in individuals with CAI [8], in addition to superior rehabilitation progress compared to resistance training over that same span [10]. Alternative balance-centric programs, such as the Progressive Hop-to-Stabilization (PHSB) and Single-Limb Balance (SLB) programs, have similarly shown improvements in several subscales (i.e., activities of daily living and sports) belonging to the Foot and Ankle Ability Measure, Star Excursion Balance Test performance, and joint position sense for dorsiflexion, plantar flexion, and inversion [11]. While the majority of existing literature supports the use of balance training in young adults and athletes, there are several studies that have noted either no significant improvements in balance or a limited effect not reflected in all balance measures [12,13,14]. These findings may be attributed to methodological inconsistencies, as some of these studies incorporated static or dynamic balance measures exclusively rather than a comprehensive balance of the two [6]. Therefore, proper assessment of the effects of balance training should include measures of both static and dynamic balance.

The slackline, an alternative to well-established balance training modalities, has been a focus of recent investigation. Slackline training requires an individual to balance while standing, walking, or performing other acts of movement while on a narrow nylon ribbon tied between two anchor points [15]. The slackline itself is subject to rapid mediolateral movements, which challenges an individual to maintain their postural control on a small, moveable base of support [16]. In a meta-analysis conducted by Donath et al., the effects of slackline training have been evaluated in children, adults, and seniors [17]. The main findings of this review include significant improvements in balance performance tasks related to slacklining, such as static slackline standing time, but limited transfer effects to untrained balance tasks. Indeed, interpretations should be taken with caution due to the variations in training duration, frequency, and programming. It remains to be determined if balance enhancements associated with slackline balance training can translate to balance tasks unrelated to slacklining.

Currently, our understanding of how slackline training affects elite caliber athletes remains limited. Preliminary investigation has been conducted in male Division I soccer players [18], as well as in female basketball players [19]. Both of these respective studies employed a cross-training approach, integrating their 6-week slackline balance training programs into their sport-specific training regimens. Positive effects on jump performance, acceleration, and postural control were identified in male soccer players, while improvements in countermovement jump performance and postural stability were observed in female basketball players. However, the applicability of these findings to athletes belonging to different sport disciplines has yet to be determined. In addition to the paucity of research examining the effects of slackline training in high level athletes, the slackline itself has several limitations. Despite its versatility in- and outdoors, the set up for the slackline requires stable anchor points and a sufficient amount of space for full extension of the slackline. Consideration of spacing is necessary, especially given that longer slacklines have a lower rate of turning, which subsequently limits activation of the semicircular canals [20], which are vital components of the vestibular system implicated in balance. The semicircular canals contain endolymph, which, when displaced by angular head movement, activates sensory hair cells that transmit signals to the brain in order to maintain equilibrium [21]. Recently, miniaturized versions of the slackline have been created that mitigate the previous limitations of the traditional slackline. The portability and compact features of these smaller slacklines can be especially beneficial for group- or team-based training. However, investigation is warranted to determine if slackline training with these more compact modalities can meaningfully affect balance.

To our knowledge, no randomized controlled studies have examined the effects of slackline training in American football players. Compromised balance can increase the risk of noncontact lower extremity injuries within these athletes [22], demonstrating the need for injury-preventative measures that can enhance or preserve balance. Here, we investigate the effects of an 8-week slackline training program, executed via a portable slacklining board, compared to a conventional balance training (CBT) program and control group (CON) receiving no balance training on static and dynamic balance in Division I American football players.

## 2. Materials and Methods

### 2.1. Study Design

**Figure 1:**
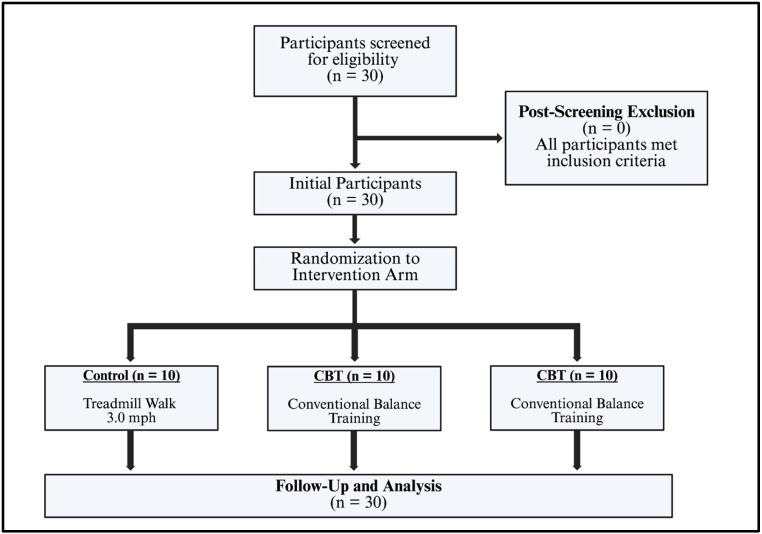
CONSORT diagram. Made with Biorender (2025).

This study was an 8-week, single-blind, randomized controlled trial employing a parallel research design. Participants were assigned in equal proportions (*n* = 10) to one of three groups within an off-season strength and conditioning program (Figure 1): intervention groups utilizing either a portable slackline balance board (SLT) or conventional balance training (CBT), and a control group that did not receive any balance training (CON). A neutral investigator, uninvolved in participant recruitment, performed the randomization utilizing an online-generated random number program. Allocation concealment was achieved using consecutively numbered envelopes. The participants trained three times per week for eight weeks (totaling 24 sessions), with each session lasting between 45–60 min. An eight-week trial was chosen to ensure adequate training adaptation from both research groups. Participants were instructed to abstain from additional balance training during the study period to prevent confounding variables. All assessments and training sessions were conducted at an off-site training facility under the supervision of a staff member who is nationally certified by the National Strength and Conditioning Association and possesses 15 years of experience.

### 2.2. Participants

Thirty male NCAA Division I American football athletes from several Southern California colleges participated in this study. Participants were required to be 18–25 years old, attended strength and conditioning sessions at least weekly for the previous two years, and were actively participating as players in an NCAA Division I American football program. The exclusion criteria involved significant medical conditions such as musculoskeletal, cardiovascular, metabolic, pulmonary, or other disorders that could limit exercise capacity or increase the risk of adverse cardiovascular events during exercise. All exploratory participants provided written informed consent while ethical approval was obtained from the University of California, Los Angeles (UCLA) (IRB: 11-003190). Off-site participants provided written informed consent and single IRB approval (sIRB: BRANY, NY, USA). Research practices were conducted in accordance with the ethical principles documented in the Declaration of Helsinki. Sample size of *n* = 30 was calculated based on a priori power analysis using results reported from an unpublished exploratory study using six collegiate-aged males of similar design in our research laboratory assuming *a* = 0.05 and *B* = 0.20.

### 2.3. Conventional Balance Training (CBT) and Slackline Balance Training (SBT) Equipment

**Figure 2:**
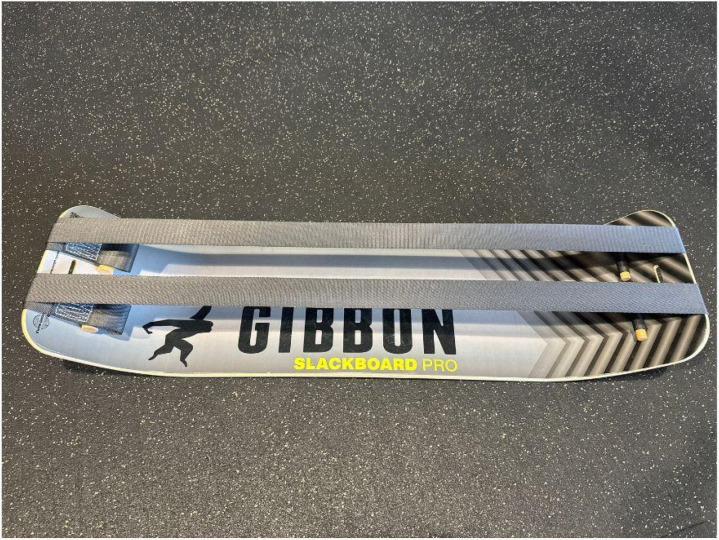
Portable slackline board used for slackline balance training.

Conventional balance training (CBT) included the use of wobble boards (multi-directional), BOSU balls employing both the dome and platform sides and inflatable cushion discs and/or a soft, closed-cell foam pad placed under the feet. Slackline balance training (SLT) utilized the GiBoard (Gibbon, Stuttgart, Germany), which is a portable balance trainer designed to offer the core advantages of slacklining in a more accessible and indoor-friendly format. It features a wooden board platform weighing 3.4 kg and measuring 1.07 m by 0.30 m, with a short slackline-style webbing suspended between two fixed points (Figure 2).

### 2.4. Supervised 8-Week Progression-Based Balance Training Program

During the two-month, thrice-weekly strength and conditioning program, which lasted 45–60 min per session, balance exercises were incorporated as distinct sessions of 15–20 min each within each session (i.e., 15–20 min of each 45–60 min training session was dedicated to the assigned intervention for this study). The control group participated in the same training regimen as the intervention groups; however, instead of the balance training component, they performed treadmill walking at a speed of 3.0 mph. Adherence, recorded as the proportion of training sessions attended and completed out of the total scheduled sessions, was tracked by a research associate that supervised the training. To ensure proper blinding of the participants, training for each group with their respective intervention was completed in separate areas. Participants were strictly confined to their training area to avoid potential unblinding and were instructed not to discuss their training with any of the other participants. A research administrator collected all of the equipment following the completion of the balance training session and hidden from apparent view.

The proprietary strength and conditioning program is designed to enhance athletic performance across various collegiate sports, including football, basketball, rugby, lacrosse, tennis, and volleyball. The primary goal established by the strength and conditioning coaches was to maximize performance in muscular strength, endurance, power, speed, and agility. Furthermore, in reference to the objective of enhancing balance, training was focused on proprioception, core stability, and dynamic balance through the implementation of various training methods. Research associates supervised workouts, recorded workout data, and ensured adherence to the training protocol.

Table 1 provides an outline of the balance training program along with a brief description of the exercises involved. The program entailed four phases, each spanning two weeks, incorporating a progression from static to dynamic to reactive balance. The difficulty increased by reducing visual input, enhancing task complexity, or incorporating dual-task activities. Both balance-training interventions (CBT and SLT) were deliberately matched to ensure that the only systematic difference between groups was the type of balance device used. To equate the induced instability stimulus, each program employed similar session frequency (thrice weekly), training duration (15–20 min), and number of exercises per session with equal work-to-rest ratios. Exercise progression was parallel between groups, advancing from bilateral to unilateral tasks and from static to dynamic movements at similar time points. Tasks in both interventions targeted comparable neuromuscular demands, including proprioceptive challenge, center-of-mass control, and reduction in the base of support. The level of postural difficulty was therefore standardized to the best possible degree across groups, with the device itself serving as the only differing source of instability. The only limitation, however, was the exercise choice differed, with the SLT group exclusively using the GiBoard and iterative exercises wherein repeated, slackline-specific movements were facilitated to achieve more refined balance.

### 2.5. Testing Procedures

After obtaining written informed consent and screening for inclusion and exclusion criteria, all participants were assessed at baseline and at the 8-week mark while adhering to identical protocols prior to each testing session. During the intervention period, they were instructed to maintain their usual daily activities and to refrain from initiating any new structured exercise or balance-training programs, including strength training, agility or plyometrics programs, or use of balance devices. Normal, non-structured physical activity (e.g., walking, routine work, recreational activity) was permitted, and participants were asked to notify study staff if their activity level changed.

To ensure consistency throughout test administration, all pre- and post-assessments took place at the same location and time of day (specifically, early evening to optimize diurnal effects on performance) conducted by the same investigator. Each participant underwent a familiarization session during which all testing procedures were practiced until confidence and proper form were achieved. The following sequence and descriptions of tests were employed.

#### 2.5.1. Anthropometrics

Anthropometric measurements were taken after an overnight fast of ≥8 h and at least 3 h of fasting prior to bioelectrical impedance analysis. Participants were instructed to remain hydrated and avoid exercising at least 2 h prior to their arrival for testing. Additionally, participants were instructed to void their bladder (i.e., urinate) before being weighed and prior to undergoing bioelectrical impedance analysis. These guidelines are in accordance with the recommendations made by the American Society of Exercise Physiologists [23].

Body mass was measured using a calibrated medical scale with an accuracy of ±0.1 kg, and height was determined using a precision stadiometer (Seca, Hanover, MD, USA; accuracy ± 0.01 m). In addition to fasting and voiding instructions, participants were instructed to remove unnecessary clothing and accessories before weighing, taking height measurements, and undergoing bioelectrical impedance analysis. Body fat percentage was measured using a validated octipolar, multi-frequency, multi-segmental bioelectrical impedance analyzer (InBody Co., Seoul, Republic of Korea) [24]. After investigators explained the procedure, the participants stood upright with their feet on two metallic footpads while holding handgrips with both hands. The instrument measured resistance and reactance using proprietary algorithms.

For balance assessments, limb length was measured bilaterally from the anterior superior iliac spine to the inferior border of the lateral malleolus, with the participant in a relaxed supine position, using a cloth tape. Lower limb length measurements were taken in order to normalize modified Star Excursion Balance Testing reach distance to leg length [25]. This measure was facilitated in order to ensure that differences in body size did not confound balance performance on the mSEBT.

#### 2.5.2. Assessment of Balance Performance

Static balance performance was measured using a force plate system while participants stood on their dominant (right) leg. The sway path was evaluated under three different conditions: standing on solid ground, balancing on a soft mat (Balance-pad, Airex), and standing on a balance wobble board (Kübler Sport). The overall sway path length (in millimeters) was measured using a force plate (EasyBase–Force Plate System; Meloq; Sweden). Technical information for this system can be found at the following URL (https://meloqdevices.com/products/forceplates-easybase?srsltid=AfmBOop86EewkNFg8OFpd_WdszNJqilYkr93mfY5PZleXYnfDhRr0qMY (accessed on 15 December 2025)). The sway path length was calculated as the total length of the trajectory of the center of pressure, summed from point-to-point Euclidean distances [26]. All balance assessments followed standard operating procedures. Three trials per device, each lasting 20 s, were recorded. Participants rested for 10 s between trials.

Dynamic balance performance was assessed using the modified Star Excursion Balance Test (mSEBT), which has been previously validated as a reliable assessment of dynamic balance [25]. Since the SEBT requires reaching in eight directions and is therefore more time consuming [27], we elected to utilize the mSEBT to assess reaching ability in the anterior, posteromedial, and posterolateral directions. The participant stood on one leg, positioning the great toe at the origin line and a line perpendicular to the origin between the second and third toes; this foot position remained consistent across all testing directions according to previously published standards. While keeping their hands on their hips, the athlete reached out with the opposite limb in each direction. Each participant executed four practice reaches and three recorded reaches in each test direction. The maximal reach distance (measured in centimeters) was determined by the distance achieved with the great toe. A trial was considered invalid and repeated if the participant lifted or moved the stance foot, transferred weight to the reach foot, lost balance, or took their hands off their hips at any point during the reach. This procedure was duplicated while standing on the opposite limb, with the order of limb testing being randomized. Normalized reach distances, as percentages, were adjusted according to the length of the stance limb, and a composition score was determined by averaging normalized reach distances across three directions. The average of the three trials was used for analysis of each outcome measure [28].

### 2.6. Statistical Analysis

Descriptive statistics are presented as median and interquartile range (IQR). The IQR is represented as a single value, which is equivalent to the numerical difference between the 25th and 75th percentiles [29]. The IQR represents the spread of the middle half of the data in a manner that is less susceptible to the influence of outliers [30]. A lower IQR indicates less variability in the central 50% of the data, whereas a higher IQR points to the middle 50% of the data being more spread out. Data deviated significantly from normality per Shapiro–Wilk tests (*p* < 0.05). Given that data was not normally distributed, the non-parametric counterparts of parametric tests (i.e., Kruskal–Wallis and Wilcoxon, One-way ANOVA and Paired *t*-test, respectively) were used for analysis. Wilcoxon signed rank-tests were utilized to compare baseline and post-intervention data within each group. A one-way Kruskal–Wallis test with post hoc Wilcoxon rank-sum tests were performed to compare delta data across all three groups. All tests were two-tailed. Statistical significance was determined beginning with α = 0.05 before the Benjamini–Hochberg method [31] was employed to control the false discovery rate. All analyses were performed using R version 4.5.0 (R Foundation for Statistical Computing, Vienna, Austria).

## 3. Results

### 3.1. Anthropometric Characterization

All participants completed the study with strong adherence to their respective training sessions. No injuries were reported by any of the participants. Average age and height for all participants was 21 ± 1.4 and 180 ± 9.7 cm, respectively. Body mass differed between baseline and post-training measurements within all three groups (Table 2). However, body mass did not significantly differ between groups either before or after training.

### 3.2. Static Balance

**Figure 3:**
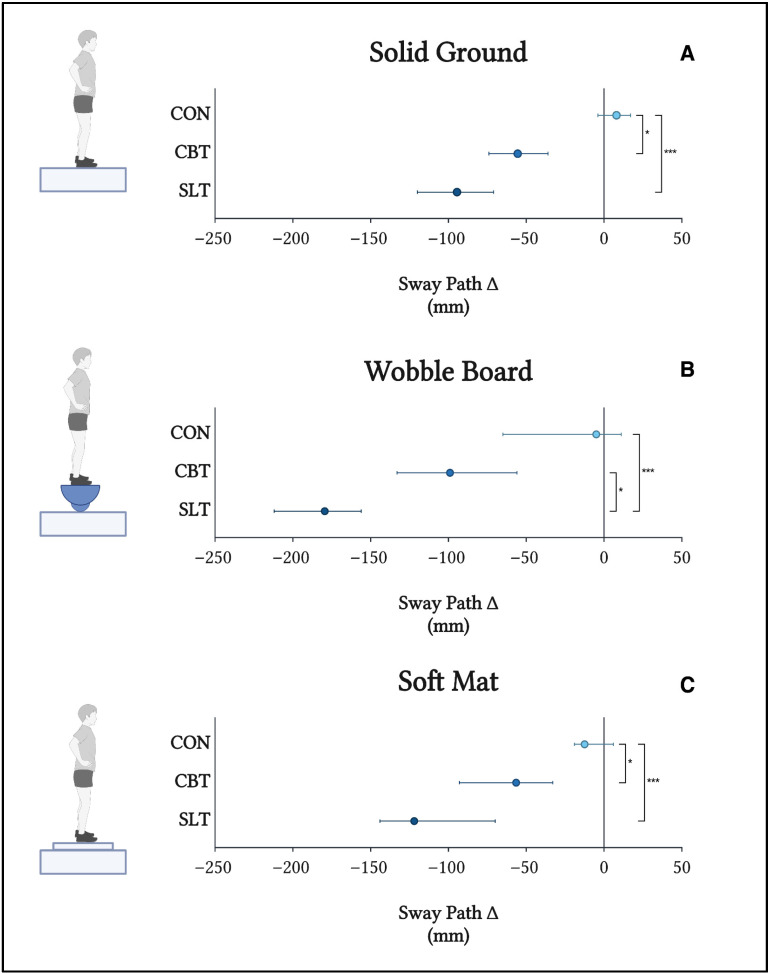
Post-training changes (∆) in sway path length (mm) on solid ground (**A**), a wobble board (**B**), and a soft mat (**C**). Comparisons of between ∆ values were made using a one-way Kruskal–Wallis test with post hoc Wilcoxon rank-sum tests. * indicates *p* < 0.05; *** indicates *p* < 0.001. The central point represents the median and error bars represent 95% CI.

Sway path length was measured on three surfaces: a firm surface, a wobble board, and a soft mat (Figure 3). Kruskal–Wallis test revealed no significant differences in sway path length at baseline between any of the three groups on the firm surface (*p* = 0.897), wobble board (*p* = 0.726), and soft mat (*p* = 0.836) conditions. Significant differences in sway path length were identified between baseline and post-training values in CBT and SLT across all three surfaces, whereas no such differences were identified in CON. Post-training sway path length was significantly lower in SLT compared to CON across all three surfaces (*p* < 0.001). Similarly, post-training sway path length in CBT was significantly lower than CON, but only in the solid ground and soft mat conditions (*p* < 0.043 and *p* < 0.012, respectively). SLT conferred a significantly greater reduction in sway path length relative to CBT only in the post-training wobble board condition (*p* < 0.031).

### 3.3. Dynamic Balance

**Figure 4:**
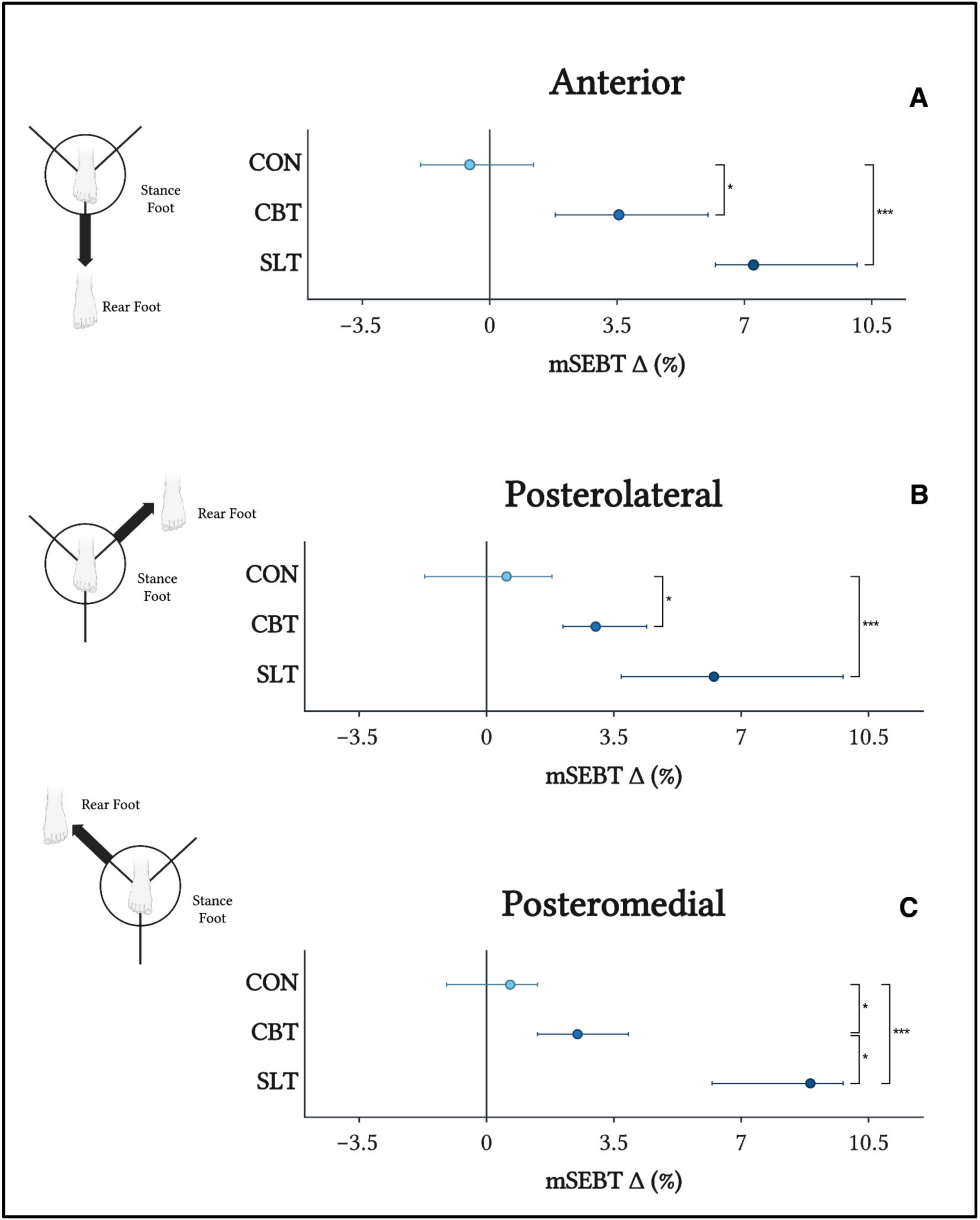
Median changes (∆) in mSEBT performance (%) in the anterior (**A**), posterolateral (**B**), and posteromedial (**C**) directions. Comparisons of mean ∆ were made using a one-way Kruskal–Wallis test with post hoc Wilcoxon rank-sum tests. * *p* < 0.05; *** *p* < 0.001. Error bars represent 95% CI.

The mSEBT measures dynamic balance across the anterior, posterolateral, and posteromedial directions, which are ultimately integrated into a normalized composite score. Changes in mSEBT performance were analyzed in each of the three directions (Figure 4). No significant differences between groups at baseline were detected via the Kruskal–Wallis test in the anterior (*p* = 0.879), posterolateral (*p* = 0.747), or posteromedial (*p* = 0.910) directions, in addition to overall composite mSEBT score (*p* = 0.913). In a similar manner to static balance measures, no significant differences between baseline and post-training mSEBT performance in any direction were detected in CON. Conversely, significant improvements in mSEBT performance across all three directions were noted in both CBT and SLT. Compared to CON, mSEBT performance in CBT (*p* < 0.05) and SLT (*p* < 0.001) was significantly better across all three directions. Notably, mSEBT performance in SLT was superior to CBT only in the posteromedial direction.

**Figure 5:**
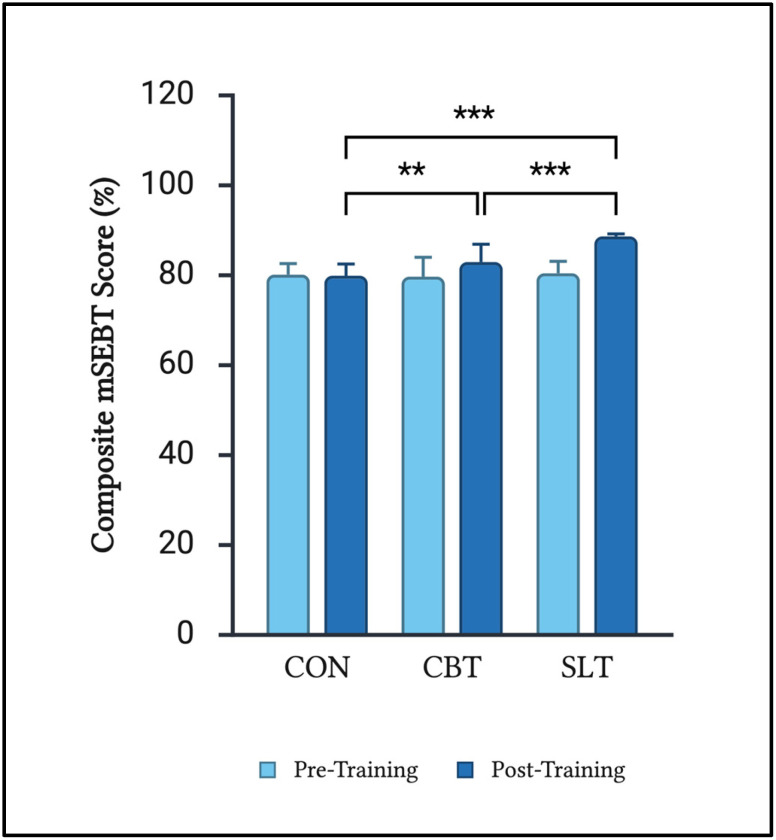
Median mSEBT normalized composite scores (%). ** *p* = 0.033; *** *p* < 0.001. Error bars represent 95% CI.

Further analysis of the integrated composite scores was conducted (Figure 5). Comparing baseline and post-training values revealed differences in composite scores in the CBT and SLT groups, but not in CON. Notably, CBT and SLT both produced significantly higher post-training composite mSEBT scores compared to CON (*p* = 0.033 and *p* < 0.001, respectively). However, post-training composite mSEBT scores corresponding to SLT were greater than CBT (*p* < 0.033).

## 4. Discussion

The primary aim of this study was to investigate the effects of an 8-week slackline training (SLT) program compared to a conventional balance training (CBT) program in Division I American football players. The SLT and CBT groups both demonstrated improvements in static and dynamic balance, measured via force plate sway path and modified Star Excursion Balance Test (mSEBT) performance, respectively. Balance enhancements were significantly greater in those who underwent SLT compared to CBT in regard to the wobble board condition, in addition to the posteromedial direction of the mSEBT. Furthermore, composite mSEBT scores were significantly higher in SLT relative to CBT. Conversely, no changes in either balance metric were observed in the control group (CON). These results are the first to demonstrate that American football players at the collegiate level may benefit from slackline balance training.

Previous investigation concerning slackline training across different demographics has yielded conflicting results. A 12-week slackline training program implemented in young soccer players was associated with improvements in balance measured by the balance error scoring system and SEBT [15]. These balance enhancements, however, were similar in magnitude to those linked to a traditional balance training program. In another study, kinematic analysis following a supervised four-week slackline training program in healthy adults found better single leg postural stability on both stable and perturbed surfaces in those who participated in slackline training [32]. However, several studies have found that slackline training effects do not translate beyond slackline-related tasks, whether that be in children [33], adults [34], or seniors [35,36]. In these contexts, benefits attributed to slackline training are limited to measures directly associated with slackline performance (e.g., single- and double-limb slackline standing time).

Speculation as to why transfer effects of slackline training remain elusive has led to neurological probing. Giboin et al. evaluated the effects of six weeks of slackline training in healthy adults on their performance of a trained and untrained balance task while monitoring neuroplasticity via the H-reflex method [37]. Magnetic resonance imaging (MRI) scans following the training period revealed elevated activation in brain regions responsible for planning, executing, and correcting motor movement, including the cerebellum, thalamus, and basal ganglia. Interestingly, despite these augmented activation patterns, performance on the untrained balance task was still not improved. H-reflex amplitude, a measure of spinal excitability, was also significantly reduced during task execution. Decreased H-reflexes while slacklining are believed to reduce reflex-mediated joint movement, which can adversely affect balance-related performance [38]. Taken altogether, these findings suggest that slackline training is associated with unique slackline- and task-specific neurological alterations that fail to generalize indiscriminately to untrained balance tasks.

Several factors may explain the static and dynamic balance improvements observed in the present study, the first of which being the SLT program’s structure. Similar to other SLT programs, the present study also used a cross-training approach. Since SLT was completed prior to the more rigorous, energy-demanding exercises corresponding to the other offseason training program, the confounding effects of fatigue were likely circumvented. During states of fatigue, individuals are susceptible to compromised reflexes [39,40] and dynamic joint control [41,42]. In relation to balance training, training while fatigued can adversely affect balance outcomes. Keller et al. demonstrated that individuals who performed balance training during fatigued states, following a high intensity interval training routine, showed less postural stability compared to those who completed their balance training in an unfatigued state [26]. Thus, we posit that performing SLT at the beginning of their training sessions may have played a role in maximizing the balance benefits of the training.

However, the results may be further explained by the program’s training intensity and volume. Neural, strength, and mass adaptations accompanying training, especially in high level athletes, are subject to plateau, which ultimately necessitates novel stimuli in order to elicit further training adaptations [43]. Variety has been hypothesized to help overcome adaptation plateaus [44], which can be implemented into training programs through new training exercises, modalities, and/or oscillating intensity. Given that none of the participants in this study had prior slackline training experience, it is possible that this unique form of balance training was indeed sufficiently stimulating for these participants. Previous investigation into the metabolic demands of slacklining supports this notion. In a cohort of healthy adults participating in a five-day slackline training program with 15 min daily training sessions, the average energy expenditure measured via metabolic equivalents (METs) was approximately 6.0 ± 0.7 METs [45]. According to the 2018 Physical Activity Guidelines Advisory Committee’s Scientific Report, an MET value ≥ 6.0 indicates physical activity of a vigorous intensity [46]. Given that this SLT program utilized an 8-week training period with sessions of a similar duration, the training volume at this intensity may have been optimal for physically challenging these elite athletes and eliciting balance improvements.

Dynamic balance ability is an essential attribute in collegiate American football players, especially as it relates to injury risk. In a cohort of 59 collegiate American football players, players who scored below an 89.6% on the SEBT, a measure of dynamic balance, demonstrated a three-fold increase in their likelihood of experiencing a noncontact lower extremity injury [22]. It should be noted that not all measures of dynamic balance, such as the Lower Quarter Y-Balance Test (YBT-LQ), are accurate predictors of injury risk [47]. Nevertheless, mitigating injury risk through balance training holds considerable potential. At the high school level, a balance pad training intervention held prior to and throughout the duration of a competitive football season was associated with a 77% reduction in noncontact ankle injuries [48]. The cohort in this study [48] included high school football players characterized by a “low-, moderate-, or high-risk” of ankle injury based on their body mass index and injury history. Given the relative success of this intervention in decreasing injury incidence, this highlights the possibility for the SLT utilized in the present study to be leveraged for a similar purpose.

Interpretations and practical applications of the findings from this study should be taken with respect to several limitations. The exclusive use of male collegiate American football players limits generalizability to other demographics, such as athletes belonging to different sport disciplines, those who are primarily sedentary, women, etc. The football players in this study also played skill positions, such as running back, defensive back, and wide receiver; this therefore limits interpretation in a position-dependent manner and may not extend to other positions, such as offensive or defensive linemen. To this end, the sample size may limit the statistical robustness of these findings; substantiation is warranted in larger samples. Additionally, given that the interventions investigated in this study were implemented alongside another offseason-specific training program, it is plausible that the effects observed may be partially attributed to this other program. The duration of the intervention training sessions was subject to slight variations, which must also be taken into account. Practical interpretations of these results indicate that eight weeks of SLT can produce static and dynamic balance enhancements, as demonstrated by reduced force plate sway path and mSEBT performance. However, future research should determine if the balance enhancements associated with SLT can transfer to untrained balance tasks, if SLT can reduce injury incidence, and if these findings can be repeated in athletes of other sports. Another potential avenue for exploration would be determining if SLT can influence sport-specific performance metrics, such as speed, agility, and power.

## 5. Conclusions

The present study identified balance improvements associated with an 8-week slackline training (SLT) program, compared to a conventional balance training (CBT) program and control (CON) program, in Division I American football players. The SLT and CBT groups both demonstrated improvements in static and dynamic balance, measured via force plate sway path and modified Star Excursion Balance Test (mSEBT) performance, respectively. These results are the first to demonstrate that American football players at the collegiate level may benefit from slackline balance training. Further research should determine if balance improvements in these metrics correlate with injury risk, in addition to investigating these programs in athletes belonging to other sports disciplines.

## Figures and Tables

**Table 1:** Balance training programs.

Conventional Balance Training (CBT) Regimen	Slackboard Balance Training (SLT) Regimen
**Week 1–2** *Wobble Board*: Double-leg stance (3 sets of 30 s) with eyes open, alternating weight shifts from forward-back/side-to-side *Bosu Ball* (flat side downwards): Squat (2 sets of 10 reps), static balance with double-leg stance (3 sets of 30 s) *Cushion Disc*: Seated single-leg lift with arms crossed (3 sets of 30 s hold per leg), Standing double-leg balance with eyes open	**Week 1–2** Single-leg static hold (30 s × 3): eyes open, weight shift forward-back/side-to-side Reverse lunge (3 sets of 10): non-slackline knee soft touch floor Squat (3 sets of 10): non-slackline foot shoulder-width stance, lift leg off floor to knee-bend
**Week 3–4** *Wobble Board*: Single-leg stance (2 sets of 20 s hold per leg) with eyes open, medicine ball toss to a partner or wall while standing (1 set of 10 reps) *Bosu Ball* (flat side downwards): Squat with medicine ball toss (2 sets of 10 reps), step on/off with alternating legs (2 sets of 10 reps per leg) *Cushion Disc*: Standing single-leg hold with head rotations to the left and right (2 sets of 5 rotations per direction on each leg), lateral lunges on/off with alternating legs	**Week 3–4** Reverse lunge w/perpendicular board & upper body twist (3 sets of 10) Walk across (3 sets of 10): forward and reverse Reverse lung crossover (3 sets of 10): after knee touch leg crossover board to pike
**Week 5–6** *Wobble Board*: Single-leg balance with alternations between sets with either medicine ball tosses or eyes closed (2 sets of 30 s per leg), forward lunges with back foot on the board (2 sets of 10 reps per leg) *Bosu Ball* (flat side downwards): Lateral jumps onto Bosu Ball (2 sets of 8 reps per side), *Cushion Disc*: Tandem stance with light resistance band arm pulls (2 sets of 8 reps per arm), Clock reach on single-leg stance with reaches in 3 different directions	**Week 5–6** Single-leg static hold (30 s × 3): eyes closed, weight shift forward-back/side-to-side Reverse lunge (3 sets of 10): non-slackline knee soft touch floor with eyes closed Squat (3 sets of 10): non-slackline foot shoulder-width stance, lift leg off floor to knee-bend with one eye closed
**Week 7–8** *Wobble Board*: Reaction step from stable two-leg stance and stepping into direction called out (3 sets of 8 reps), catch and squat with light medicine ball while balancing (3 sets of 8 reps) *Bosu Ball* (flat side upwards): Squat (2 sets of 6 reps), alternating step-ups with knee drive (3 sets of 6 reps per leg) *Cushion Disc*: Single-leg hop to disc and hold (3 sets of 10 s hold per leg), double-leg stance with eyes closed while balancing and experiencing light perturbations	**Week 7–8** Reverse lunge w/perpendicular board & upper body twist (3 sets of 10): react to called out direction Walk across (3 sets of 10): forward and reverse reacting to called out direction Side shuffle (3 sets of 10): keep torso rigid, bend at hips reacting to called out direction

**Table 2:** Pre- and post-training parameters represented as median values (IQR) corresponding to body mass, as well as static and dynamic balance. Wilcoxon signed rank-tests were facilitated to compare pre- and post-training values. * indicates *p* < 0.05; CON = Control; CBT = Conventional Balance Training; SLT = Slackline Balance Training.

	CON			CBT			SLT		
	Baseline	Post- Training	*p*-Value	Baseline	Post- Training	*p*-Value	Baseline	Post- Training	*p*-Value
Body Mass	86.6 (8.5)	88.6 (9.1)	0.009 *	86.3 (8.9)	87.8 (8.5)	0.009 *	85.9 (8.5)	88.1 (10.2)	0.009 *
Sway Path Length (mm) Firm Surface	779 (34.3)	794 (41.3)	0.330	788 (35.3)	727 (52.3)	0.011 *	778.0 (51.0)	687.0 (84.8)	0.007 *
Sway Path Length (mm) Wobble Board	914 (168.5)	904 (151.8)	0.570	971 (33.5)	883 (48.5)	0.007 *	988 (66.5)	799 (43.0)	0.009 *
Sway Path Length (mm) Soft Mat	879 (87.0)	878 (102.5)	0.111	845 (53.5)	789 (48.5)	0.009 *	829 (87.3)	745 (76.8)	0.007 *
mSEBT (%) Anterior	60 (3.3)	61 (1.3)	0.590	60 (3.4)	64 (3.6)	0.007 *	61 (1.1)	68 (1.4)	0.007 *
mSEBT (%) Posterolateral	82 (5.8)	82 (2.7)	0.883	83.0 (5.0)	87 (4.6)	0.007 *	84 (6.7)	90 (1.9)	0.009 *
mSEBT (%) Posteromedial	97 (4.3)	97 (5.0)	0.579	99 (5.1)	101 (4.8)	0.009 *	99 (4.0)	107 (3.8)	0.009 *
mSEBT (%) Composite	80 (3.9)	80 (3.0)	0.910	80 (3.1)	83 (3.1)	0.009 *	80 (3.1)	89 (1.0)	0.007 *

## Data Availability

The data set of the present study is available from the corresponding author upon reasonable request.

## Abbreviations

The following abbreviations are used in this manuscript:

SLT	Slackline Balance Training
CON	Control
CBT	Conventional Balance Training
mSEBT	Modified Star Excursion Balance Test
IQR	Interquartile Range
